# A 60 Hz uniform electromagnetic field promotes human cell proliferation by decreasing intracellular reactive oxygen species levels

**DOI:** 10.1371/journal.pone.0199753

**Published:** 2018-07-16

**Authors:** Kiwon Song, Sang Hyeon Im, Yeo Jun Yoon, Hui Min Kim, Hae June Lee, Gwan Soo Park

**Affiliations:** 1 Department of Biochemistry, College of Life Science & Biotechnology, Yonsei University, Seoul, Korea; 2 Department of Electrical Engineering, Pusan National University, Pusan, Korea; Consiglio Nazionale delle Ricerche, ITALY

## Abstract

Previously, we showed that exposure of human normal and cancer cells to a 6 mT, 60 Hz gradient electromagnetic field (EMF) induced genotoxicity. Here, we investigated the cellular effects of a uniform EMF. Single or repetitive exposure to a 6 mT, 60 Hz uniform EMF neither induced DNA damage nor affected cell viability in HeLa and primary IMR-90 fibroblasts. However, continuous exposure of these cells to an EMF promoted cell proliferation. Cell viability increased 24.4% for HeLa and 15.2% for IMR-90 cells after a total 168 h exposure by subculture. This increase in cell proliferation was directly correlated with EMF strength and exposure time. When further incubated without EMF, cell proliferation slowed down to that of unexposed cells, suggesting that the proliferative effect is reversible. The expression of cell cycle markers increased in cells continuously exposed to an EMF as expected, but the distribution of cells in each stage of the cell cycle did not change. Notably, intracellular reactive oxygen species levels decreased and phosphorylation of Akt and Erk1/2 increased in cells exposed to an EMF, suggesting that reduced levels of intracellular reactive oxygen species play a role in increased proliferation. These results demonstrate that EMF uniformity at an extremely low frequency (ELF) is an important factor in the cellular effects of ELF-EMF.

## Introduction

Extremely low frequency (ELF) electromagnetic fields (EMFs) are produced when electricity is generated and transmitted, such as in transmission lines, railways, and electrical home appliances [1]. EMFs of 0–300 Hz are defined as ELF-EMFs. We are exposed daily to 50–60 Hz ELF-EMFs produced by most electrical home appliances [1, 2].

Several epidemiological studies have suggested that ELF-EMF increases the risk of developing cancer, including leukemia, brain, and breast cancers [3–5]. Thus, there have been concerns regarding the latent biological risk of ELF-EMFs. Some cell-based studies reported that 50–60 Hz of ELF-EMFs induce DNA double-strand breaks (DSBs), activation of cell cycle checkpoints, chromosomal instability, and apoptosis. For example, 14 μT EMFs of 60 Hz induced apoptosis in mouse testicular germ cells, 100 μT EMFs of 50 Hz arrested the cell cycle at G1 in human SH-SY5Y neuroblastoma cells, 1 mT EMFs of 60 Hz induced chromosomal instability in human fibroblasts, and 5 mT EMFs of 60 Hz led to cell death through reactive oxygen species (ROS) generation in human HL-60 promyelocytic leukemia cells [6–9]. Thus, the International Agency for Research on Cancer (IARC) classified ELF-EMFs as a group 2B carcinogen in 2002.

In contrast, other studies using ELF-EMFs of similar intensity and frequency ranges reported that ELF-EMFs have no cellular effects due to their low energy. For example, the cellular effects of 60 Hz EMFs at 1 mT were negligible in mouse fibroblast NIH/3T3 cells, human lung fibroblast WI-38 cells, human lung epithelial L132 cells, and human mammary gland epithelial MCF10A cells [10, 11]. Moreover, a 50 Hz EMF at 2 mT had no effect on rodent brain cells [12]. Meanwhile, other studies showed that 0.5–5 mT ELF-EMFs promote cell proliferation in human epidermal stem cells, prostate cancer cell lines, HL-60 leukemia cells, rat-1 fibroblasts, and WI-38 diploid fibroblasts [13–15]. Thus, the cellular effects of ELF-EMFs remain highly controversial as their biological effects are not consistent and the cause for this discrepancy is not yet fully understood. The inconsistency of cellular effects induced by ELF-EMFs strongly suggests that there is an overlooked parameter that plays a key role in modulating its effect on cells.

Previously, we showed that a single or repetitive exposure to a gradient EMF of 60 Hz at 6 and 7 mT induces DSBs and apoptosis [16, 17]. In an attempt to interpret the discrepancy between these genotoxic effects, we dissected the factors responsible for cellular effects of a 60 Hz EMF at 6 and 7 mT and paid attention to the uniform or gradient characteristics of the EMF.

In this study, we designed a novel device that generates a uniform EMF of 60 Hz at 1–10 mT and examined its cellular effects to verify the importance of EMF characteristics. Unlike a gradient EMF of 60 Hz, a uniform 60 Hz EMF showed neither genotoxic nor apoptotic effects. Rather, it increased cell proliferation in both cancer and normal cells, strongly suggesting that uniform or gradient characteristics of ELF-EMFs play a key role in cellular responses.

## Materials and methods

### A uniform ELF-EMF-generating device

**Fig 1:**
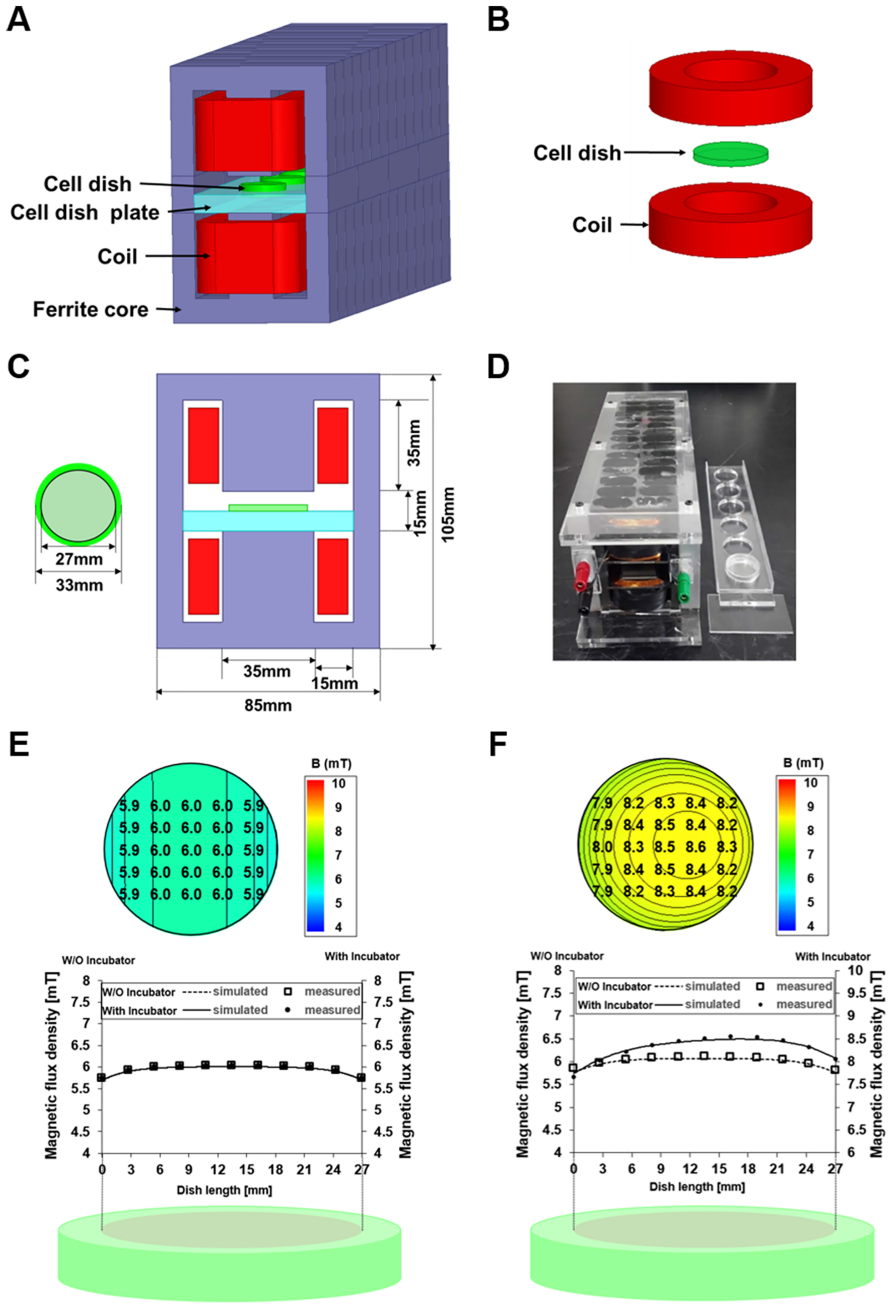
A 60 Hz EMF device generates a uniform EMF. (A) Closed-type ELF-EMF device. (B) Open-type ELF-EMF device. (C) Schematic front view and size of ELF-EMF device. (D) Manufactured EMF device. (E) Simulated and measured magnetic flux density distribution by using Maxwell 3D software and a Gauss meter in the closed-type device. (F) Simulated and measured magnetic flux density by Maxwell 3D and a Gauss meter in the open-type device.

A closed-type ELF device was designed as shown in Fig 1 and S1a Fig. To generate strong magnetic fields, a highly permeable ferrite core with a closed-type flux path was adopted. Two E-shaped cores were placed facing each other (S1b and S1c Fig). Since the magnetic resistance of this design was centered on the middle gap, the magnetic field is focused on the middle gap and may reduce the coil current. The culture plates were placed in the 15 mm center gap. The magnetic flux density inside the dish was designed to generate up to 10 mT without the coil overheating. To enable multiple exposures simultaneously, a multi-dish plate can be placed as shown in S1d Fig. For 6 dishes, 28 cores were used in this design.

Each coil is 0.4 mm in diameter and has 1,000 turns. Magnetic flux density inside the dish is easily controlled as it is proportional to the coil current. Thus, this device can generate strong and uniform magnetic fields without overheating. The magnetic field was measured by a Gauss meter (7010; F.W. Bell, Milwaukie, OR, USA) and the design parameters are summarized in Table 1. As shown in Fig 1e, the magnetic field of the plate is uniform in all 6 dishes.

**Table 1:** Design parameters for the uniform ELF-EMF device.

Parameter	Value
Length	280 mm
Coil diameter	0.4 mm
Coil turns	1,000 turns
Current	0.08 A
Material	ferrite core
Maximum magnetic flux density without overheating	10 mT

### Cell culture

Human cervical cancer HeLa and human lung fibroblast IMR-90 cells were purchased from the American Type Culture Collection (ATCC; Manassas, VA, USA). HeLa cells were cultured in high glucose-containing Dulbecco’s modified Eagle medium (DMEM; Gibco, USA) supplemented with 10% fetal bovine serum (FBS; Sigma-Aldrich, St. Louis, MO, USA) and 1% penicillin–streptomycin (Gibco). IMR-90 cells (passages 8–13) were grown in minimum essential medium (MEM; Gibco) supplemented with 10% FBS and 1% penicillin–streptomycin. Both cells were maintained at 37 °C in a humidified atmosphere containing 5% CO_2_.

### Exposure to uniform ELF-EMF

Cells (3 × 10^4^) were plated in 35 mm culture plates. After 20 h of incubation, the cells were exposed to a 60 Hz uniform ELF-EMF at 1, 3, 6, or 10 mT for different periods of time. The incubator was monitored by an NT-312 probe to maintain a temperature of 37 ± 0.5 °C.

### Cell proliferation assay

Cell viability was analyzed by spectrophotometric measurement of mitochondrial dehydrogenase activity using 3-(4,5-dimethylthiazol-2-yl)-2,5-diphenyltetrazolium bromide (MTT) [18]. Following incubation with 0.5 mg/mL MTT, the formazan products were dissolved in 1 mL DMSO, transferred to 96-well plates, and measured using a microplate reader (SOFTmax PRO 4.0; Molecular Devices, CA, USA). The relative viability was expressed as the optical density (OD) value at 570 nm of the exposed cells relative to that of unexposed control.

Cell number was counted with a hemocytometer (Neubauer; Marienfeld-Superior, Germany) following the manufacturer’s protocol.

### Western blot analysis

Cells were harvested, washed with cold phosphate buffered saline (PBS; Gibco-BRL), and lysed in whole cell lysis buffer containing 50 mM Tris (pH 7.4), 1 mM EDTA, 1 mM EGTA, 1% Triton X-100, 0.1% β-mercaptoethanol, and 0.1% SDS. Histones were extracted using 0.25 M HCl containing 10% glycerol and neutralized with 1 M NaOH. Proteins (30 μg of whole cell lysate or 10 μg histone) were resolved by 10% SDS-PAGE and electro-transferred to polyvinylidene difluoride membrane prior to the incubation with the primary antibody to detect the protein of interest. Anti-sera against β-actin (1:3000), histone H3 (1:3000), phospho-histone H3 (1:3000), CDK4 (1:1000), p-Erk1/2 (1:1000), Erk1/2 (1:2000), p-Akt (1:1000), Akt (1:2000) (Cell Signaling Technology, Danvers, MA, USA), and γ-H2AX (1:3000, Abcam, MA, USA) were used. Relative protein expression was normalized to β-actin and H3 using ImageJ software (National Institutes of Health, MD, USA).

### Intracellular ROS detection

HeLa cells (3×10^4^) seeded on 35 mm culture plates with gelatin-coated glass coverslips were incubated for 20 h and continuously exposed to 60 Hz ELF-EMFs for 72 h and 168 h. Cells were then incubated with Hank’s balanced salt solution (HBSS) containing 5 μM 5(6)-carboxy-2′,7′-dichlorodihydrofluorescein diacetate (carboxy-H_2_DCFDA; Invitrogen, CA, USA) for 30 min at 37 °C in the dark. Nuclei were co-stained with Hoechst 33342 (Invitrogen). Coverslips were washed twice with cold PBS and mounted with HBSS. Cells were imaged using an Axio Imager A2 (Carl Zeiss, Jena, Germany) equipped with an AxioCam Hrc CCD camera (Carl Zeiss) and then analyzed with the AxioVision software (Carl Zeiss). Excitation/emission wavelengths were 492–495/516–527 nm for carboxy-H_2_DCFDA and 350/461 nm for Hoechst 33342. Fluorescence signals of carboxy-H_2_DCFDA were obtained with fixed 5,000 ms exposures.

### Glucose oxidase treatment

After 18 h of cell seeding, cell culture medium was removed, cells were washed with PBS, and then replenished with culture medium containing 0.1 mU/mL of glucose oxidase (GOx).

### Flow cytometry analysis

For cell cycle analysis, cells were detached and harvested at each time point with trypsin-EDTA (Gibco-BRL), washed with cold PBS, and fixed in 70% cold ethanol for 2 h. Cells were washed in 2% FBS containing cold PBS, resuspended in PBS with 100 μg/mL RNase A (Bio Basic Inc., ON, Canada) for 30 min at 37 °C, and then stained with 10 μg/mL propidium iodide (PI; Sigma-Aldrich) for 15 min at room temperature in the dark.

To measure intracellular ROS levels, cells harvested by trypsin were stained with 5 μM carboxy-H_2_DCFDA in HBSS for 30 min in a CO_2_ incubator and washed twice with cold PBS. Flow cytometry was performed using a BD FACSCalibur (BD Biosciences, NJ, USA), analyzed with BD CellQuest Pro software (BD Biosciences), and then data was analyzed using Flowing software (v 2.5.1; http://www.uskonaskel.fi/flowingsoftware).

### Statistical analysis

All experiments were performed at least three times. Results are expressed as the mean ± standard deviation (SD). Statistical analysis was performed using GraphPad Prism (v 6.01; GraphPad Software Inc., CA, USA). P-values < 0.05 were considered statistically significant, while P-values > 0.05 were not significant (ns).

## Results

### An E-shaped core generates a uniform ELF-EMF

To examine the cellular effects of a uniform 60Hz EMF exposure on human cells, a closed-type EMF device was designed to generate a strong and uniform magnetic field (Fig 1A); the parameters are detailed in Table 1.

Our device has many advantages compared with the commonly used HelmHoltz type open device, as illustrated in Fig 1B. Owing to the highly permeable magnetic core, strong magnetic fields can be produced by a relatively small coil current. If the magnetic resistance of the core and the field fringing on the pole side are neglected, the magnetic flux density inside the culture plate can be derived as follows,

$$\vec{B}(t)=\frac{\mu_{0}\cdot N}{g}\cdot I(t)\cdot {\hat{a}}_{y},$$ (1)

Where *μ*_*0*_ is the permeability, *g* is the gap between the two poles, *N* is the number of coil turns, and *I(t)* is the coil current. At a frequency of 60 Hz, the current and magnetic flux density of the culture plates are time-varying.

To generate the same magnitude of 6 mT magnetic flux density in the culture plates, the closed-core coil only needs a current of 24 mA whereas the open type Helm-Holtz coil needs 144 mA, even though the number of coil turns in both devices are 1,000. As the heat of the coil is proportional to the square of the current, the Helm-Holtz coil may cause a heating problems inevitably during experiments.

The most important advantage of our device is magnetic shielding from the external environment. During experiments, the culture plate is located inside an incubator that is covered with iron. In the case of an open-type device, even if the magnetic field distribution is designed uniformly, field is changed by the highly permeable iron incubator. However, since the closed structure is used in our device, there is no influence by the external environment and a uniform magnetic field distribution is maintained. In such circumstances, although field distributions are designed to produce a uniform field, it is inevitably affected by the highly permeable iron incubator.

The spatial distributions of magnetic flux densities of culture plates in closed-core type and open-type Helm-Holtz devices are shown in Fig 1E and 1F. The magnitude and distribution of the magnetic flux density of culture plates in the closed-core type device is invariant inside the incubator. The variation of magnetic field is within 0.1 mT, which keeps the uniformity deviation under 1.67%. However, for culture plates in the open type Helm-Holtz, the magnitude changes and distributions are distorted up to 8.2%, which means a difference between the maximum and minimum value is 2.51 mT, and the consistency of the cell experiment could be prevented (Fig 1F). Therefore, the following experiments were conducted using a closed-type device that has been validated to apply a uniform magnetic flux density.

### A single exposure to a uniform ELF-EMF neither induces DNA damage nor affects cell viability in cancer and normal cells

**Fig 2:**
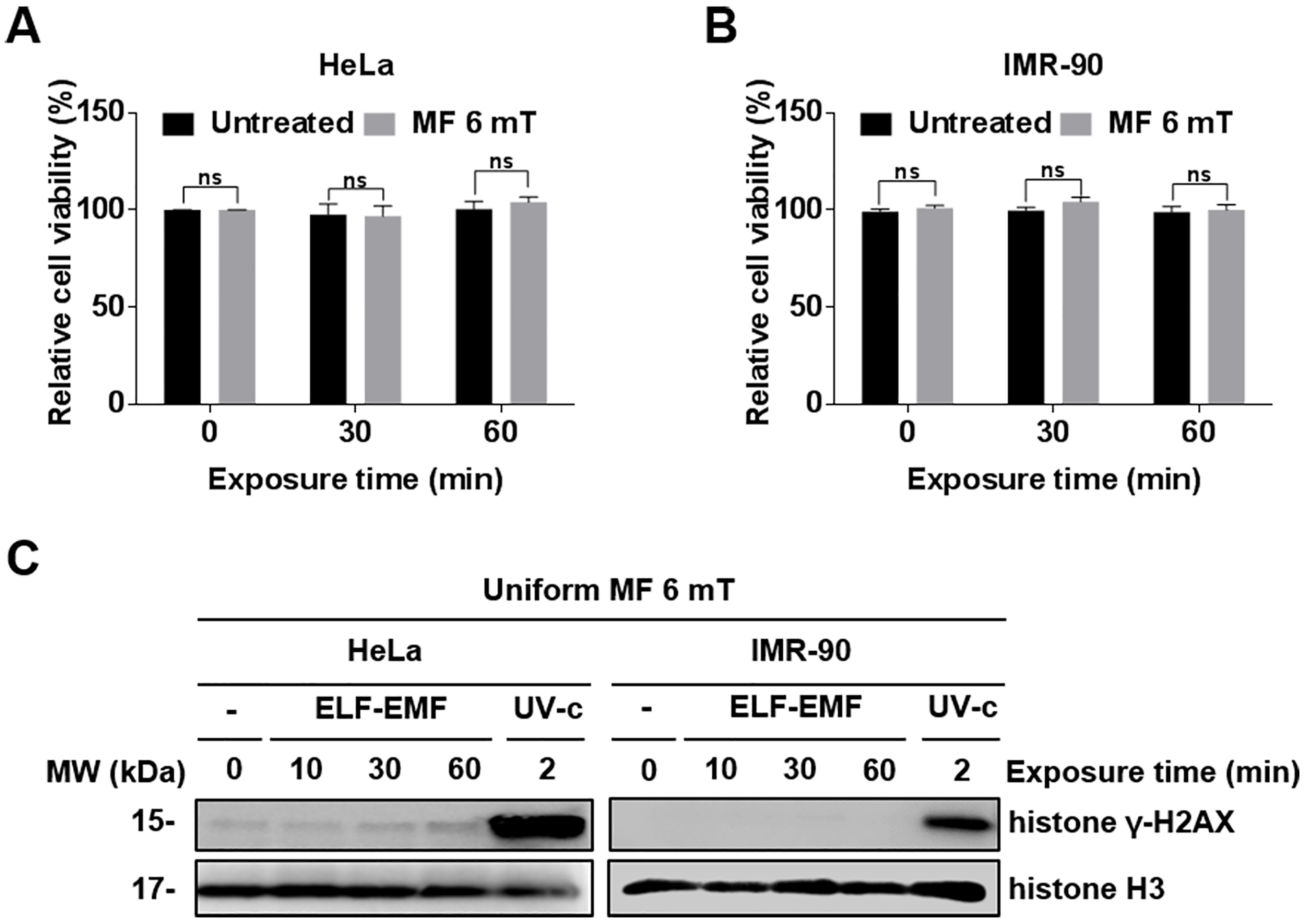
Cellular effects of a single exposure to a uniform EMF of 6 mT. (A, B) HeLa and IMR-90 cells were exposed to an EMF of 6 mT for 0, 30, and 60 min. Cell viability was measured by MTT assays and evaluated as a percentage relative to the viability of unexposed cells (0 min). Values are presented as the mean ± SD (n = 3) and P-values were determined by two-way ANOVA with the Bonferroni correction. Values of *P < 0.05, **P < 0.01, ***P < 0.001, and ****P < 0.0001 were considered statistically significant, and P > 0.05 was considered statistically not significant (ns). (C) γ-H2AX was assessed by western blot analysis. Histone H3 was used as a loading control and cells exposed to ultraviolet (UV) (100 J/m^2^) were used as positive controls for DNA damage.

A 60 Hz uniform ELF-EMF is used throughout the study unless otherwise stated. Using the newly fabricated device, we examined if the exposure of human cells to a uniform EMF induced acute cellular effects similar to a gradient EMF. The cervical cancer HeLa cell line and human lung fibroblast IMR-90 cells were used to assess whether the effects of ELF-EMF exposure is different between cancer and normal cells. After both cell types were exposed to an ELF-EMF, the cell viability was estimated by MTT assays. In both HeLa and IMR-90 cells, a single exposure to an EMF for 30 and 60 min did not affect viability (Fig 2A and 2B). Previous studies, including ours, reported that ELF-EMFs act as genotoxic stress and can induce DSBs [19, 20]. Thus, we examined DNA damage in HeLa and IMR-90 cells after exposure to a uniform ELF-EMF by detecting γ-H2AX. γ-H2AX is phosphorylated H2AX and a DSB marker. No phosphorylation of H2AX was observed (Fig 2C). These data indicate that a single exposure of both cell types to an EMF did not have any detectable effects.

### Repetitive exposure to a uniform ELF-EMF neither induces DNA damage nor affects cell proliferation

**Fig 3:**
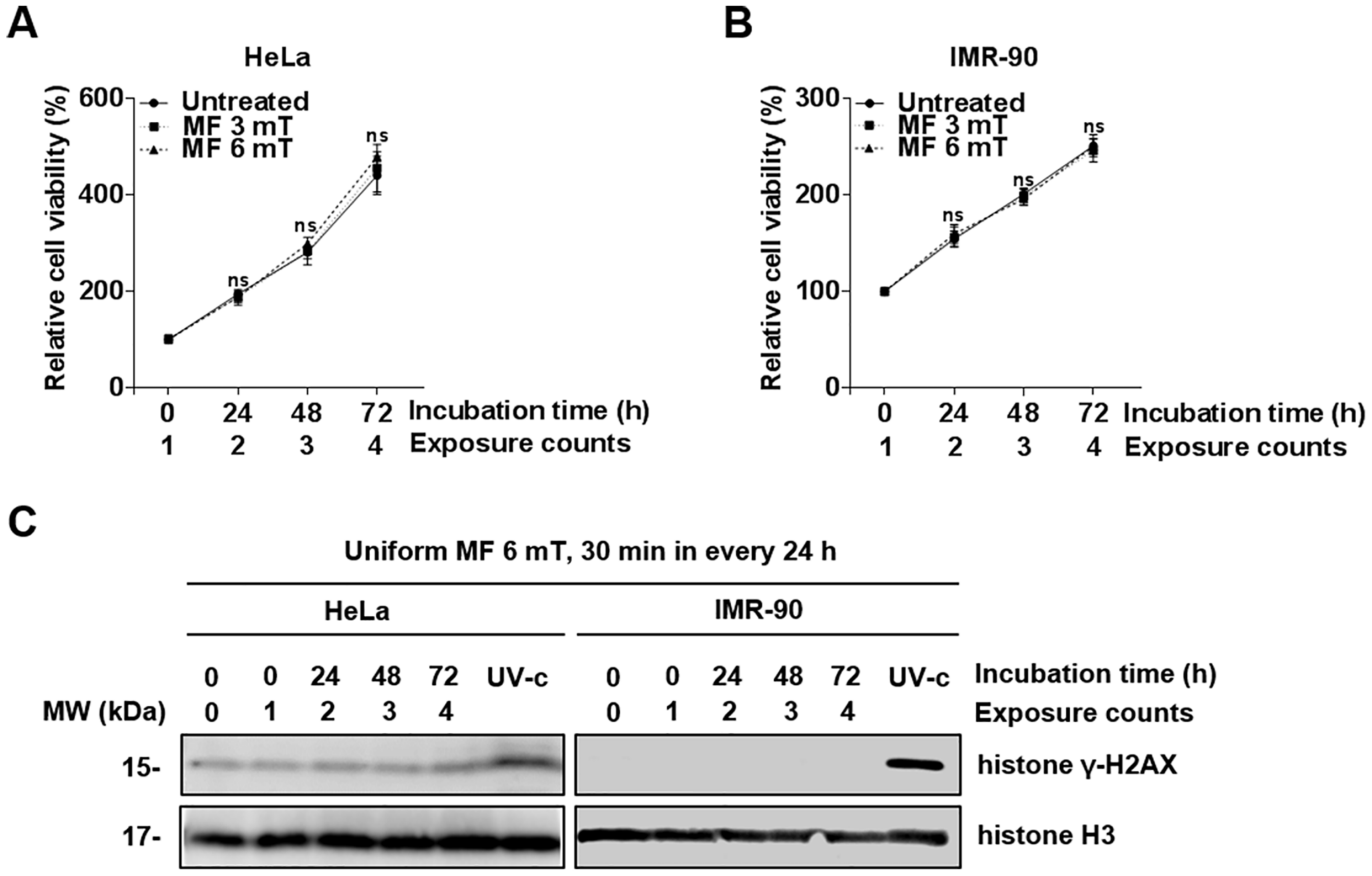
Cellular effects of repetitive exposures to a uniform EMF at 3 and 6 mT. (A, B) HeLa and IMR-90 cells were exposed to a repetitive EMF at 3 or 6 mT for 30 min every 24 h for 3 days. After incubation, cell viability was assessed by MTT assays and evaluated as a percentage relative to the viability of unexposed cells (0 h). Values are presented as the mean ± SD (n = 3) and P-values were determined by two-way ANOVA with the Bonferroni correction. P > 0.05 was considered statistically not significant (ns). (C) γ-H2AX was detected by western blot analysis. N.C., unexposed negative control. Histone H3 was used as a loading control and cells exposed to UV (100 J/m2) were used as positive controls for DNA damage.

We then further investigated the cellular effects on human cells of repetitive exposure to an EMF at 3 and 6 mT. When both HeLa and IMR-90 cells were exposed to an ELF-EMF at 3 and 6 mT for 30 min every 24 h for 72 h, there was no decrease in cell viability (Fig 3A and 3B). Furthermore, γ-H2AX levels were similar to that of the negative control (Fig 3C). Instead, we observed a slight increase in cell viability of HeLa cells that were repetitively exposed to an ELF-EMF at 6 mT, but this increased viability was not statically significant. To clarify the increased viability by repetitive exposures to ELF-EMFs, we exposed HeLa and IMR-90 cells to an ELF-EMF of 6 mT for 30 min in 30 min intervals, 8 times per day for 3 days, and then assessed cell viability every 24 h for 3 days. No statistically significant increase in cell viability was observed in both HeLa and IMR-90 cells (S2A and S2B Fig). These results demonstrate that repetitive exposure of HeLa and IMR-90 cells to an EMF neither induced any genotoxic effects nor enhanced cell proliferation.

### Continuous exposure to uniform ELF-EMFs promotes cell proliferation and its effect is reversible

**Fig 4:**
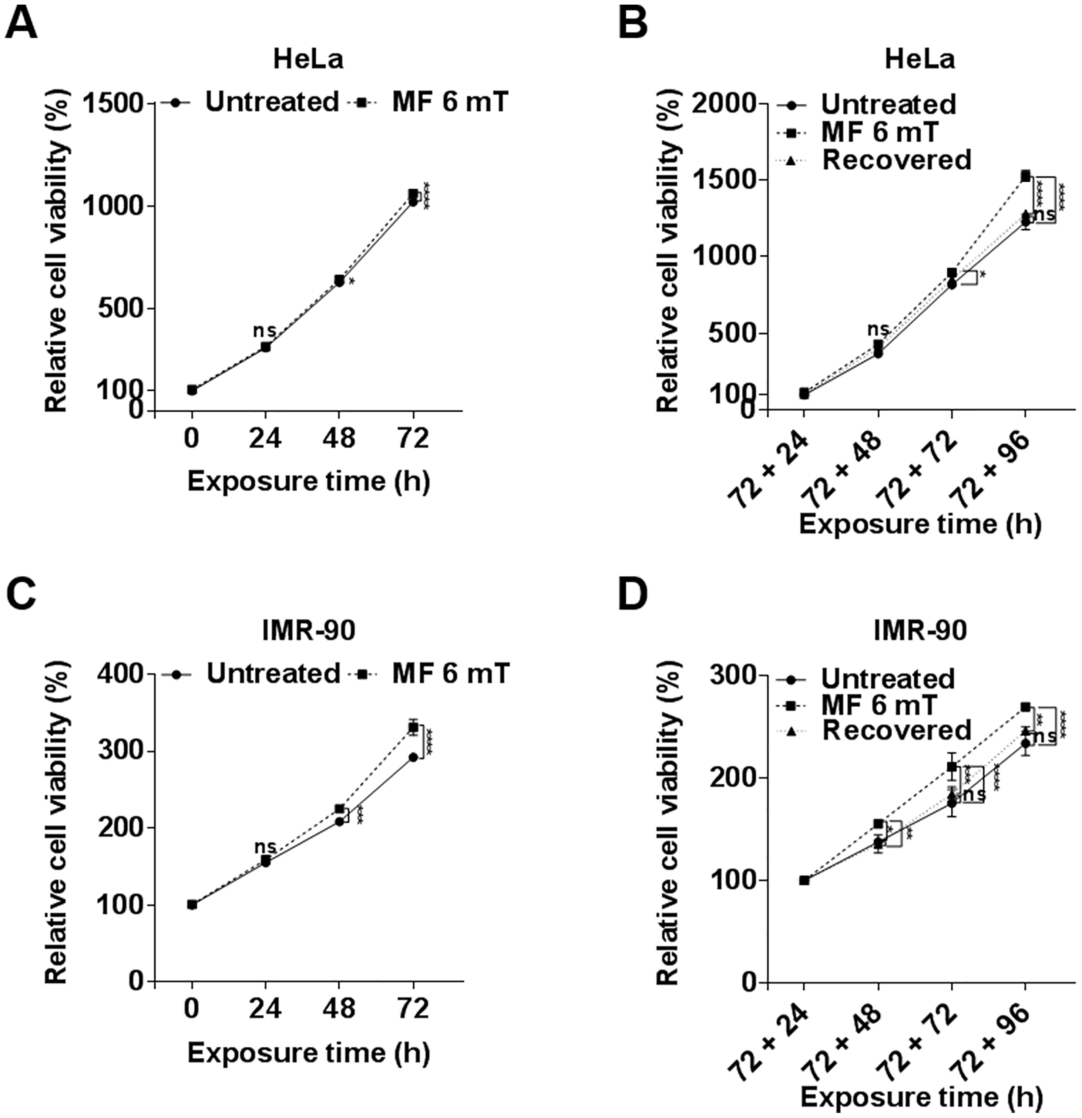
Continuous exposure to a uniform ELF-EMF promotes cell proliferation in HeLa and IMR-90 cells. (A, C) HeLa and IMR-90 cells were exposed continuously to a 6 mT ELF-EMF for up to 72 h. Cell viability was assessed by MTT assays every 24 h and evaluated as a percentage relative to the viability of unexposed cells (0 h). (B, D) After 72 h of ELF-EMF exposure, HeLa and IMR-90 cells were detached and further subcultured in an ELF-EMF of the same strength for up to 96 h. For the recovered group, cells exposed for 72 h were detached and subcultured without any further EMF exposure. Cell viability was assessed by MTT assays every 24 h and relative cell viability was evaluated as to the viability of 72 + 24 h cells. Data were plotted as the mean ± SD (n = 3). P-values were determined by two-way ANOVA with the Bonferroni correction. Values of *P < 0.05, **P < 0.01, ***P < 0.001, and ****P < 0.0001 were considered statistically significant, and P > 0.05 was considered statistically not significant (ns).

Since single and repetitive exposures to an EMF at 3 or 6 mT neither induced DNA damage nor decreased cell viability, we examined the cellular effects of a continuous exposure to an ELF-EMF of 6 mT in both HeLa and IMR-90 cells. When exposed to this EMF for up to 72 h, the viability of HeLa and IMR-90 cells increased 4.1% and 13.3%, respectively, compared with EMF-unexposed groups (Fig 4A and 4C). These observations suggest a trend of increased cell proliferation in response to a continuous exposure to ELF-EMFs. To clarify this effect, HeLa and IMR-90 cells already exposed to an ELF-EMF for 72 h were detached and subcultured in an EMF at 6 mT for an additional 96 h. We observed a marked increase in viability of cells that were grown and subcultured under an EMF for a total of 168 h; viability increased 24.4% in HeLa and 15.2% in IMR-90 cells compared with unexposed cells. We also confirmed this increased viability by hemocytometer cell counting (S3A and S3C Fig). These observations demonstrate that a continuous exposure to an ELF-EMF activates cell proliferation in both HeLa and IMR-90 cells.

Surprisingly, when HeLa and IMR-90 cells that had been exposed to EMFs for 72 h were further incubated for 96 h without EMF exposure (a recovered group), the increased cell viability trend was no longer observed and the viability was similar to that of unexposed control cells (Fig 4B and 4D). These observations suggest that the proliferative effect of a continuous exposure to an EMF is reversible and temporary. We also confirmed that this increased viability was reversible by hemocytometer cell counting (S3B and S3D Fig).

We also examined the proliferative effect of an EMF by continuous exposure to different magnetic field strengths. HeLa and IMR-90 cells were continuously incubated for 72 h under an EMF at 1, 6, or 10 mT. At 1, 6, and 10 mT, cell viability increased 6.5%, 12.1%, and 21.2% in HeLa cells, respectively, and by 1.6%, 9.5%, and 16.3% in IMR-90 cells, respectively, compared with viability of unexposed cells (S4A and S4B Fig). Enhanced increase in cell proliferation was observed with stronger magnetic fields, strongly suggesting that proliferative effects induced by EMFs depend on the magnetic field strength.

### Continuous exposure to a uniform ELF-EMF activates cell cycle progression without perturbing individual cell cycle stages

**Fig 5:**
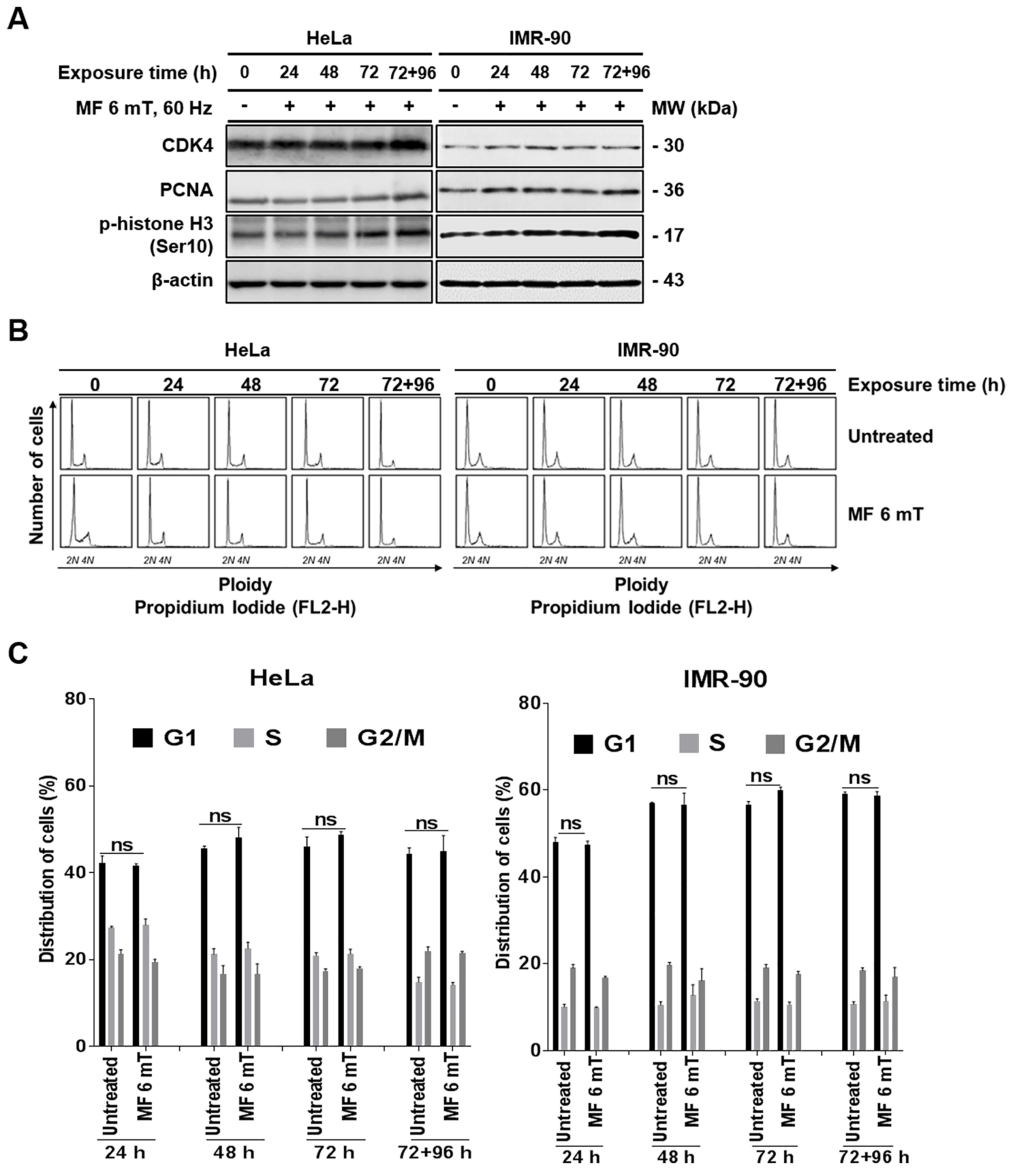
Continuous exposure to a uniform ELF-EMF activates cell cycle progression without perturbing individual cell cycle stages. HeLa and IMR-90 cells were exposed to an EMF at 6 mT for 72 h and then subcultured for an additional 96 h under the same ELF-EMF. (A) After continuous exposure, the expression of cell cycle stage markers (CDK, PCNA, and p-H3) was detected by western blotting. Indicated time denotes the total time cells were exposed to ELF-EMFs. β-actin was used as a loading control. (B) Cell cycle distribution of HeLa and IMR-90 cells exposed to an ELF-EMF was determined by flow cytometry using PI staining. For each flow cytometric analysis, 10,000 cells were counted and plotted. (C) From the flow cytometric data obtained from three independent experiments in (B), the average percentage of cells in each stage of the cell cycle was plotted as a bar graph. Values are presented as the mean ± SD (n = 3) and P-values were determined by two-way ANOVA with the Bonferroni correction. P > 0.05 was considered statistically not significant (ns).

Since we observed increased cell proliferation induced by an ELF-EMF in both HeLa and IMR-90 cells, we next examined the expression of cell cycle progression markers. Cyclin-dependent kinase 4 (CDK4) was used as a marker for G1 progression, proliferation cell nuclear antigen (PCNA) for DNA synthesis in S phase, and phosphorylated histone H3 (p-H3) for G2/M. The expression levels of CDK4, PCNA, and p-H3 increased in a time-dependent manner in cells subjected to an EMF when compared with unexposed control (Fig 5A). Increased expression of these markers specific for different cell cycle stages suggests that continuous exposure to an EMF may not activate a specific cell cycle process. To confirm, we analyzed the distribution of HeLa and IMR-90 cells in different cell cycle stages by flow cytometry every 24 h (Fig 5B). After comparing with untreated control cells, we did not detect any significant changes in cell cycle distribution of HeLa and IMR-90 cells after exposure to an ELF-EMF (Fig 5B and 5C). Altogether, these observations suggest that continuous exposure to an EMF slightly accelerates overall cell cycle progression for increased proliferation without dysregulation of a specific cell cycle stage.

### Continuous exposure to a uniform EMF decreases intracellular ROS levels

We questioned how exposure to an EMF increased cell proliferation. Several previous studies reported that ELF-EMFs can alter intracellular ROS levels [21–23]. ROS are constantly generated in cells by metabolic redox reactions. A minor increase in intracellular ROS levels may induce cell proliferation through promoting signaling cascades [24]. Supplementing with antioxidants also increases cell proliferation by reducing intracellular ROS levels [25, 26]. Thus, we first investigated if activated proliferation induced by uniform EMFs is due to alterations of intracellular ROS levels.

**Fig 6:**
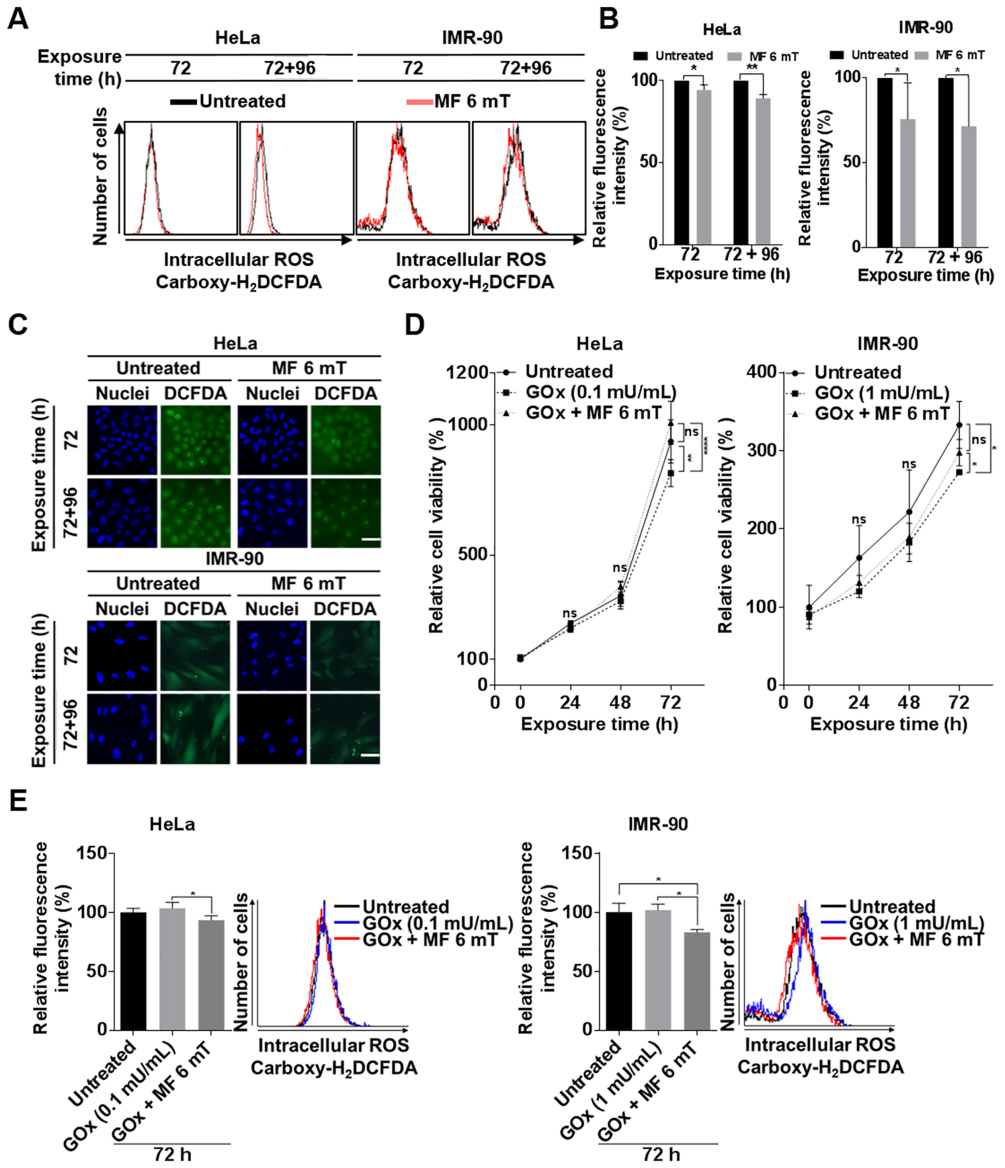
Continuous exposure to a uniform ELF-EMF reduces intracellular ROS levels in HeLa and IMR-90 cells. HeLa and IMR-90 cells were exposed to an EMF at 6 mT for 72 h and further subcultured under the same ELF-EMF for an additional 96 h. Indicated time denotes the total time cells were exposed to uniform ELF-EMFs. (A) Intracellular ROS levels were monitored by carboxy-H_2_DCFDA fluorescence using flow cytometry. For each flow cytometric analysis, 10,000 cells were counted and plotted. The data are shown as overlapping histogram plots. (B) From data obtained in (A), the relative fluorescence intensity of EMF-exposed cells and untreated control was quantified from the geometric mean of fluorescence intensity in 10,000 cells and then plotted as the mean ± SD (n = 3). (C) Representative fluorescence microscopy images. Scale bar = 50 μm. Carboxy-H_2_DCFDA (green) represents intracellular ROS levels. Cell nuclei are stained with Hoechst 33342 (blue). (D) Cells were incubated with 0.1 mU/mL GOx alone or GOx under a uniform EMF of 6 mT for 72 h. Cell viability was measured by MTT assays every 24 h and evaluated as a percentage relative to the viability of untreated cells (0 h). Data were plotted as the mean ± SD (n = 3). (E) After 72 h incubation of (D), intracellular ROS levels were monitored by carboxy-H_2_DCFDA fluorescence through flow cytometry. For each flow cytometric analysis, 10,000 cells were counted and plotted (left) as the mean ± SD (n = 3). The data are shown as overlapping histogram plots (right). (B, D, E) P-values were determined by two-way ANOVA with the Bonferroni correction. Values of *P < 0.05, **P < 0.01, ***P < 0.001, and ****P < 0.0001 were considered statistically significant, and P > 0.05 was considered statistically not significant (ns).

Human cells uptake non-fluorescent carboxyl-H_2_DCFDA, which is then oxidized by intracellular ROS to emit a bright green fluorescent signal. We monitored the fluorescence by flow cytometry in HeLa and IMR-90 cells incubated under an EMF of 6 mT for up to 72 h and 96 h further by subculture. The relative intensity of fluorescence slightly decreased in an exposed time-dependent manner in HeLa and IMR-90 cells exposed to an EMF compared with unexposed control (Fig 6A). When the relative fluorescence intensity was quantified in comparison with unexposed cells, it was on average reduced to 6.7%, and 11.6% in HeLa and 24.5% and 27.9% in IMR-90 after 72 and 72+96 h of incubation by EMF exposure, respectively. (Fig 6B). To confirm flow cytometry data, we also observed the intensity of fluorescence in HeLa cells by fluorescence microscopy; consistent with Fig 6A, intracellular ROS levels decreased in cells exposed to an EMF (Fig 6C). These observations suggest that one of the main causes of increased proliferation is the time-dependent reduction in intracellular ROS levels.

To confirm that increased proliferation of HeLa and IMR90 cells by continuous exposure to an EMF is mainly the result of decreased ROS levels, we exposed these cells to an ELF-EMF in the presence of GOx, which continuously produces low concentrations of intracellular H_2_O_2_ and reduces cell viability [27]. If continuous exposure to an ELF-EMF decreased intracellular ROS levels, then exposure to an ELF-EMF would mitigate the anti-proliferative effects of GOx. The viability of HeLa and IMR-90 cells treated with GOx decreased after 72 h of incubation compared with untreated control (Fig 6D). As expected, however, we detected that cell viability slightly increased in HeLa and IMR-90 cells exposed to an ELF-EMF in the presence of GOx after 72 h of incubation, compared with the cells treated only with Gox (Fig 6D). Using carboxyl-H_2_DCFDA and flow cytometry, we examined the relative intracellular ROS levels of HeLa and IMR-90 cells treated with GOx under the EMF for 72 h in comparison with those of HeLa and IMR-90 cells only treated with GOx. Relative to untreated HeLa and IMR-90 cells, fluorescence intensity decreased in cells treated with 0.1 mU/mL GOx under the EMF, while cells only incubated with GOx showed a slight increase in fluorescence intensity (Fig 6E). Altogether, these results strongly support that continuous exposure to an ELF-EMF reduces intracellular ROS levels.

### Continuous exposure to a uniform EMF increases phosphorylation of Akt and Erk1/2

**Fig 7:**
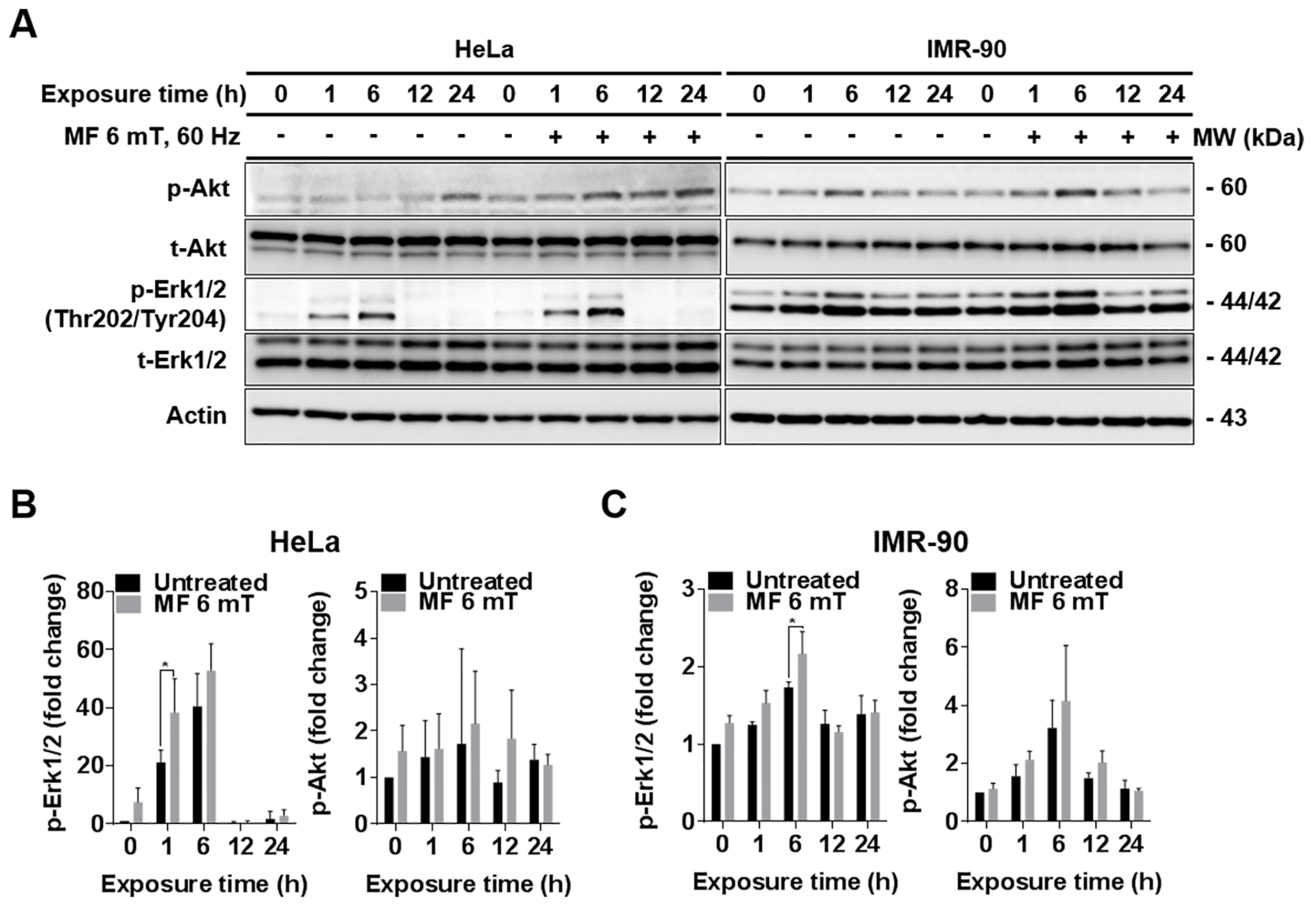
Continuous exposure to a uniform ELF-EMF increases phosphorylation of Akt and Erk1/2 in HeLa and IMR-90 cells. (A) HeLa and IMR-90 cells were continuously exposed to a 6 mT ELF-EMF for 1, 6, 12, 24 h and then expression levels of Erk1/2, p-Erk1/2, Akt, and p-Akt were detected by western blot analysis. β-actin served as a loading control. (B, C) Calibration histograms were used to quantify p-Akt and p-Erk1/2 levels from three independent experiments including (A). Relative protein expression levels of total Akt (t-Akt) and Erk1/2 (t-Erk1/2) were first normalized to β-actin and then p-Akt and p-Erk1 levels were normalized to t-Akt and t-Erk1/2. Values are presented as the mean ± SD (n = 3) and P-values were determined by two-way ANOVA with the Bonferroni correction. Values of *P < 0.05, **P < 0.01, ***P < 0.001, and ****P < 0.0001 were considered statistically significant, and P > 0.05 was considered statistically not significant (ns).

To further confirm that increased proliferation of HeLa and IMR90 cells by continuous exposure to an EMF is mainly due to decreased ROS levels, we examined the intracellular ROS-mediated signaling pathways that control cell growth. Several studies have shown that reduced intracellular ROS levels by antioxidants increase phosphorylation of Erk1/2 and Akt [28, 29]. Consistent with this, we observed phosphorylation of Akt and Erk1/2 in HeLa and IMR-90 cells exposed to an EMF for 1, 6, 12, and 24 h by western blot analysis. In comparison with untreated cells incubated at the same durations, the levels of phospho-Akt and phospho-Erk1/2 were slightly increased in both HeLa and IMR-90 cells exposed to an ELF-EMF at 1 and 6 h (Fig 7A–7C). These results suggest that an ELF-EMF induces cell proliferation by activating Akt and Erk1/2 (Fig 7A–7C).

## Discussion

We are constantly exposed to ELF-EMFs of 50–60 Hz in our daily lives; thus, there is a growing public interest in the thermal effects of ELF-EMFs. However, the biological effects of ELF-EMFs remain highly controversial. Some studies reported adverse effects, such as DNA damage and cell death, whereas others suggested an application of ELF-EMFs in cell differentiation and even wound healing [6, 13, 30, 31]. Since the cellular effects of ELF-EMFs are inconsistent and previous studies mainly focused on frequency and intensity, we questioned if an ELF-EMF parameter that plays a key role in modulating cellular effects had been overlooked.

In this study, we focused on the uniformity of ELF-EMFs applied to cells as a key parameter. Most studies on ELF-EMFs claimed to have used a device generating ‘uniform ELF-EMFs’ when the device was placed inside of a CO_2_ incubator. However, the incubator and especially its inner chambers are usually made of metal, thus the EMF applied to the cells would not be uniform (Fig 1f and S1f Fig). We designed a novel ELF-EMF generator that maintains EMF uniformity in the cell incubator (S1e Fig). Our results showed that exposure to uniform ELF-EMFs neither induced DNA damage nor cell death, but rather promoted cell proliferation in both primary IMR-90 fibroblasts and cancer HeLa cells in a time- and magnetic field strength-dependent manner. The increased proliferation induced by ELF-EMFs was reversible and did not modify cell cycle distribution of the cells. Notably, the increase in cell proliferation was accompanied by a slight decrease in intracellular ROS levels, which increased Akt and Erk1/2 phosphorylation.

Finely-tuned regulation of intracellular ROS levels is known to be important in the control of proliferation and differentiation in mammalian cells (for a review, see Sauer *et al*. [32]). Excessive intracellular ROS levels is cytotoxic and leads to senescence and cell death [33], whereas a mild reduction in intracellular ROS levels induced by antioxidants increases cell proliferation by activating Akt and Erk1/2 [28, 29]. Interestingly, some studies suggest that exposure to ELF-EMFs increases or decreases intracellular ROS levels, thereby inducing various cellular effects. A 50 Hz ELF-EMF exposure for 3 or 24 h was reported to enhance ROS generation and DNA damage in rat-1 fibroblasts [15]. Moreover, continuous exposure to a 50 Hz EMF of 0.6 mT increased intracellular ROS levels and DNA damage in the prostate cancer cell line DU-145 [34]. On the other hand, exposure of a human microglial cell line to a 50 Hz EMF of 1 mT attenuated cell death by reducing intracellular ROS levels [21]. Exposure to 0.11 mT ELF-EMFs increased the activities of superoxide dismutase isoenzymes and glutathione peroxidase, which are antioxidants to catalyze reduction of intracellular ROS, thereby decreasing intracellular ROS levels in murine squamous cells [22]. Modification of intracellular ROS levels by exposure to ELF-EMFs can also explain our previous results that demonstrated a genotoxic effect in HeLa and IMR-90 cells exposed to a gradient 60 Hz ELF-EMF at 6 or 7 mT [16, 17]. Kirson *et al*. [35] reported that intracellular dielectrophoresis occurs when gradient ELF-EMFs are applied to cells, moving polar molecules toward higher field intensities and thus generating ROS. The increased levels of p-Akt and p-Erk1/2 by uniform ELF-EMFs observed in this study might also explain the cell proliferation induced by ELF-EMFs reported by other researchers [36–41].

In the present study, we showed that uniform ELF-EMF exposures led to an increase in cell proliferation accompanied by a slight decrease in intracellular ROS levels. Since the proliferative effect of a uniform ELF-EMF was reversible and sensitive, the change in intracellular ROS levels by ELF-EMFs was not expected to be dramatic. Increased proliferation must be due to a subtle and sustained antioxidant effect by uniform ELF-EMFs. Thus, we only detected a slight decrease in intracellular ROS levels by micrographs and flow cytometry in cells exposed to a uniform ELF-EMF as shown in Fig 6. We also indirectly proved that this increased proliferation effect was due to a decrease in ROS levels by demonstrating a decrease in cell proliferation after exposure to a uniform ELF-EMF in the presence of GOx, which continuously produces low concentrations of intracellular hydrogen peroxide (Fig 6). This minute decrease in intracellular ROS levels as has also been demonstrated to promote proliferation of lung cancer cells when treated with low concentrations of N-acetyl-cysteine (NAC, 250 μM and 1 mM) or the soluble vitamin E analogue Trolox (25 and 100 μM) as antioxidants [26].

Altogether, this study in combination with previous reports strongly suggest that ELF-EMF exposure leads to a change in intracellular ROS levels that may result in genotoxic stress or enhanced proliferation, depending on ROS concentration and the differential sensitivity of various cells to ROS. Thus, the mechanism behind ELF-EMF exposure altering intracellular ROS levels should be further studied to elucidate the biological effects of ELF-EMFs.

The ELF-EMF device that we that we fabricated has a unique design with an E-shaped core that maintains not only uniformity of ELF-EMFs but also completely blocks any external EMFs, even geomagnetism. Cells are always exposed to geomagnetism of 0.5 Gauss. There is only one report on the cellular effects of geomagnetism which suggests that the absence of geomagnetism induced epigenetic changes in induced pluripotent stem cells from mouse fibroblasts [42]. Since geomagnetism may affect cellular physiology, the specific geomagnetism effects should be further investigated when applying ELF-EMFs to cells.

## Conclusion

The cellular effects of ELF-EMFs remain highly controversial as their biological effects are inconsistent. ELF-EMFs showed genotoxic effects and inhibited proliferation [19, 20], while also activating proliferation for wound healing [43–45]. However, the reason for this discrepancy is not yet fully understood. The inconsistency of cellular effects induced by ELF-EMFs strongly suggests that there is an overlooked parameter that plays a key role in modulating EMF effects on cells. In this study, we showed that the uniformity of ELF-EMFs is a major parameter and that exposure to a uniform ELF-EMF increases cell proliferation in human cancer and normal cells by reducing intracellular ROS levels. Since increased proliferation was observed in both normal and cancer cells, this observation may support some epidemiological studies that suggest ELF-EMFs accelerate tumor formation [3, 5, 46, 47] and the finding that ELF-EMFs may also help wound healing by activating cell proliferation [43–45]. The cellular effect of a uniform ELF-EMF should be further investigated in various human cell types to understand the overall pathological effects of ELF-EMFs.

## Supporting information

S1 Fig Core and coil of the closed-type ELF-EMF device. (A) Dimensions (mm) of the core device. (B) E-shaped ferrite core. (C) Bottom parts of the ELF-EMF device. (D) Plate for multiple dishes. (E) Variations of the flux line generated by the closed-type device in an incubator. (F) Variations of the flux line generated by the open-type device in an incubator.
(TIF)

S2 Fig The cellular effect of repetitive exposure to a uniform EMF at 6 mT. (A, B) HeLa and IMR-90 cells were exposed to an ELF-EMF of 6 mT for 30 min with 30 min intervals 8 times per day for 3 days and then cell viability was assessed by MTT assays every 24 h for 3 days. Cell viability was evaluated as a percentage relative to the viability of unexposed cells (0 h). Values are presented as the mean ± SD (n = 3) and P-values were determined by two-way ANOVA with the Bonferroni correction. P > 0.05 was considered statistically not significant (ns).
(TIF)

S3 Fig Continuous exposure to a uniform EMF promotes cell proliferation in HeLa and IMR-90 cells. (A, C) HeLa and IMR-90 cells were continuously exposed to an EMF of 6 mT for up to 72 h. Cell number was counted every 24 h with a hemocytometer. (B, D) After 72 h of exposure to the EMF, HeLa and IMR-90 cells were detached and further subcultured in a uniform ELF-EMF of the same strength for up to 96 h. For the recovered group, cells exposed for 72 h were detached and subcultured without any further EMF exposure. In each group, cell number was counted every 24 h with a hemocytometer. Data were plotted as the mean ± SEM (n = 7). P-values were determined by two-way ANOVA with the Bonferroni correction. Values of *P < 0.05, **P < 0.01, ***P < 0.001, and ****P < 0.0001 were considered statistically significant, and P > 0.05 was considered statistically not significant (ns).
(TIF)

S4 Fig A uniform EMF induces cell proliferation depending on EMF strength. (A) HeLa and (B) IMR-90 cells were exposed to an EMF at 1, 6, and 10 mT for 72 h. Cell viability was assessed by MTT assays after a 72 h exposure. Relative cell viability (the viability of exposed cells relative to unexposed cells) of an EMF at 1, 6, and 10 mT was plotted as the mean ± SD (n = 3) and P-values were determined by two-way ANOVA with the Bonferroni correction. Values of *P < 0.05, **P < 0.01, ***P < 0.001, and ****P < 0.0001 were considered statistically significant, and P > 0.05 was considered statistically not significant (ns).
(TIF)

## References

[1] Moriyama K, Yoshitomi K. Apartment electrical wiring: A cause of extremely low frequency magnetic field exposure in residential areas. Bioelectromagnetics. 2005;26(3):238–41. 10.1002/bem.20099 15768426

[2] Mezei G, Kheifets LI, Nelson LM, Mills KM, Iriye R, Kelsey JL. Household appliance use and residential exposure to 60-Hz magnetic fields. J Expo Anal Env Epid. 2001;11(1):41–9. 10.1038/sj.jea.7500145 11246800

[3] Bowman JD, Thomas DC, London SJ, Peters JM. Hypothesis—the Risk of Childhood Leukemia Is Related to Combinations of Power-Frequency and Static Magnetic-Fields. Bioelectromagnetics. 1995;16(1):48–59. 7748203 10.1002/bem.2250160111

[4] Cleary SF. A review of in vitro studies: low-frequency electromagnetic fields. Am Ind Hyg Assoc J. 1993;54(4):178–85. 10.1080/15298669391354531 8480633

[5] Wertheimer N, Leeper E. Electrical wiring configurations and childhood cancer. Am J Epidemiol. 1979;109(3):273–84. 453167 10.1093/oxfordjournals.aje.a112681

[6] Luukkonen J, Hoyto A, Sokka M, Liimatainen A, Syvaoja J, Juutilainen J, et al Modification of p21 level and cell cycle distribution by 50 Hz magnetic fields in human SH-SY5Y neuroblastoma cells. Int J Radiat Biol. 2016:1–9. 10.1080/09553002.2017.1235298 27646005

[7] Kim YW, Kim HS, Lee JS, Kim YJ, Lee SK, Seo JN, et al Effects of 60 Hz 14 mu T Magnetic Field on the Apoptosis of Testicular Germ Cell in Mice. Bioelectromagnetics. 2009;30(1):66–72. 10.1002/bem.20448 18839413

[8] Winker R, Ivancsits S, Pilger A, Adlkofer F, Rudiger HW. Chromosomal damage in human diploid fibroblasts by intermittent exposure to extremely low-frequency electromagnetic fields. Mutat Res. 2005;585(1–2):43–9. 10.1016/j.mrgentox.2005.04.013 16009595

[9] Ding GR, Nakahara T, Hirose H, Koyama S, Takashima Y, Miyakoshi J. Extremely low frequency magnetic fields and the promotion of H2O2-induced cell death in HL-60 cells. Int J Radiat Biol. 2004;80(4):317–24. 10.1080/09553000410001679802 15204708

[10] Jin YB, Choi SH, Lee JS, Kim JK, Lee JW, Hong SC, et al Absence of DNA damage after 60-Hz electromagnetic field exposure combined with ionizing radiation, hydrogen peroxide, or c-Myc overexpression. Radiat Environ Bioph. 2014;53(1):93–101. 10.1007/s00411-013-0506-5 24305851

[11] Hong MN, Han NK, Lee HC, Ko YK, Chi SG, Lee YS, et al Extremely Low Frequency Magnetic Fields Do Not Elicit Oxidative Stress in MCF10A Cells. J Radiat Res. 2012;53(1):79–86. 22302048 10.1269/jrr.11049

[12] McNamee JP, Bellier PV, Chauhan V, Gajda GB, Lemay E, Thansandote A. Evaluating DNA damage in rodent brain after acute 60 Hz magnetic-field exposure. Radiat Res. 2005;164(6):791–7. 16296885 10.1667/rr3465.1

[13] Zhang MS, Li XP, Bai LM, Uchida K, Bai WF, Wu B, et al Effects of low frequency electromagnetic field on proliferation of human epidermal stem cells: An in vitro study. Bioelectromagnetics. 2013;34(1):74–80. 10.1002/bem.21747 22926783

[14] Khavari B, Ahmadian S, Bolouri B. The effects Of 50 Hz, 0.6 mT extremely low frequency (ELF) electromagnetic field (EMF) on proliferation of the prostate cancer cell line, DU-145. Clin Biochem. 2011;44(13):S165–S.

[15] Wolf FI, Torsello A, Tedesco B, Fasanella S, Boninsegna A, D’Ascenzo M, et al 50-Hz extremely low frequency electromagnetic fields enhance cell proliferation and DNA damage: Possible involvement of a redox mechanism. Bba-Mol Cell Res. 2005;1743(1–2):120–9. 10.1016/j.bbamcr.2004.09.005 15777847

[16] Kim J, Yoon Y, Yun S, Park GS, Lee HJ, Song K. Time-varying magnetic fields of 60 Hz at 7 mT induce DNA double-strand breaks and activate DNA damage checkpoints without apoptosis. Bioelectromagnetics. 2012;33(5):383–93. 10.1002/bem.21697 22180328

[17] Kim J, Ha CS, Lee HJ, Song K. Repetitive exposure to a 60-Hz time-varying magnetic field induces DNA double-strand breaks and apoptosis in human cells. Biochem Bioph Res Co. 2010;400(4):739–44. 10.1016/j.bbrc.2010.08.140 20816755

[18] Vistica DT, Skehan P, Scudiero D, Monks A, Pittman A, Boyd MR. Tetrazolium-based assays for cellular viability: a critical examination of selected parameters affecting formazan production. Cancer Res. 1991;51(10):2515–20. 2021931

[19] Ivancsits S, Diem E, Pilger A, Rudiger HW, Jahn O. Induction of DNA strand breaks by intermittent exposure to extremely-low-frequency electromagnetic fields in human diploid fibroblasts. Mutat Res-Gen Tox En. 2002;519(1–2):1–13. 10.1016/s1383-5718(02)00109-2 12160887

[20] Lai H, Singh NP. Acute exposure to a 60 Hz magnetic field increases DNA strand breaks in rat brain cells. Bioelectromagnetics. 1997;18(2):156–65. 9084866

[21] Duong CN, Kim JY. Exposure to electromagnetic field attenuates oxygen-glucose deprivation-induced microglial cell death by reducing intracellular Ca(2+) and ROS. Int J Radiat Biol. 2016;92(4):195–201. 10.3109/09553002.2016.1136851 26882219

[22] Polaniak R, Buldak RJ, Karon M, Birkner K, Kukla M, Zwirska-Korczala K, et al Influence of an Extremely Low Frequency Magnetic Field (ELF-EMF) on Antioxidative Vitamin E Properties in AT478 Murine Squamous Cell Carcinoma Culture In Vitro. Int J Toxicol. 2010;29(2):221–30. 10.1177/1091581809352011 20335516

[23] Frahm J, Mattsson MO, Simko M. Exposure to ELF magnetic fields modulate redox related protein expression in mouse macrophages. Toxicol Lett. 2010;192(3):330–6. 10.1016/j.toxlet.2009.11.010 19913603

[24] Storz P. Reactive oxygen species in tumor progression. Front Biosci. 2005;10:1881–96. 15769673 10.2741/1667

[25] Le Gal K, Ibrahim MX, Wiel C, Sayin VI, Akula MK, Karlsson C, et al Antioxidants can increase melanoma metastasis in mice. Sci Transl Med. 2015;7(308). 10.1126/scitranslmed.aad3740 26446958

[26] Sayin VI, Ibrahim MX, Larsson E, Nilsson JA, Lindahl P, Bergo MO. Antioxidants Accelerate Lung Cancer Progression in Mice. Science Translational Medicine. 2014;6(221). 10.1126/scitranslmed.3007653 24477002

[27] Gow AJ, Branco F, Christofidou-Solomidou M, Black-Schultz L, Albelda SM, Muzykantov VR. Immunotargeting of glucose oxidase: intracellular production of H(2)O(2) and endothelial oxidative stress. Am J Physiol. 1999;277(2 Pt 1):L271–81. 10444521 10.1152/ajplung.1999.277.2.L271

[28] Ulrich-Merzenich G, Zeitler H, Panek D, Bokemeyer D, Vetter H. Vitamin C promotes human endothelial cell growth via the ERK-signaling pathway. Eur J Nutr. 2007;46(2):87–94. 10.1007/s00394-006-0636-5 17225921

[29] Song J, Park J, Oh Y, Lee JE. Glutathione Suppresses Cerebral Infarct Volume and Cell Death after Ischemic Injury: Involvement of FOXO3 Inactivation and Bcl2 Expression. Oxid Med Cell Longev. 2015. 10.1155/2015/426069 PMC4334940 25722793

[30] Pasi F, Sanna S, Paolini A, Alquati M, Lascialfari A, Corti ME, et al Effects of extremely low-frequency magnetotherapy on proliferation of human dermal fibroblasts. Electromagn Biol Med. 2016;35(4):343–52. 10.3109/15368378.2016.1138123 27254779

[31] Seong Y, Moon J, Kim J. Egr1 mediated the neuronal differentiation induced by extremely low-frequency electromagnetic fields. Life Sci. 2014;102(1):16–27. 10.1016/j.lfs.2014.02.022 24603130

[32] Sauer H, Wartenberg M, Hescheler J. Reactive oxygen species as intracellular messengers during cell growth and differentiation. Cell Physiol Biochem. 2001;11(4):173–86. 10.1159/000047804 11509825

[33] Panieri E, Gogvadze V, Norberg E, Venkatesh R, Orrenius S, Zhivotovsky B. Reactive oxygen species generated in different compartments induce cell death, survival, or senescence. Free Radic Biol Med. 2013;57:176–87. 10.1016/j.freeradbiomed.2012.12.024 23295411

[34] Khavari BAA, Shahin A Pazhang Yaghub A Bolouri Bahram A Shafizadeh Mahshid. The Effects of Extremely Low Frequency Pulsed Electromagnetic Field on Biochemical Properties of the Prostate Cancer Cell Line, DU-145. Razi Journal of Medical Sciences. 2015;22(136):0-.

[35] Kirson ED, Dbaly V, Tovarys F, Vymazal J, Soustiel JF, Itzhaki A, et al Alternating electric fields arrest cell proliferation in animal tumor models and human brain tumors. P Natl Acad Sci USA. 2007;104(24):10152–7. 10.1073/pnas.0702916104 PMC1886002 17551011

[36] Martinez MA, Ubeda A, Moreno J, Trillo MA. Power Frequency Magnetic Fields Affect the p38 MAPK-Mediated Regulation of NB69 Cell Proliferation Implication of Free Radicals. Int J Mol Sci. 2016;17(4). 10.3390/ijms17040510 PMC4848966 27058530

[37] Martinez MA, Ubeda A, Cid MA, Trillo MA. The Proliferative Response of NB69 Human Neuroblastoma Cells to a 50 Hz Magnetic Field is mediated by ERK1/2 Signaling. Cell Physiol Biochem. 2012;29(5–6):675–86. 10.1159/000178457 22613968

[38] Nie K, Henderson A. MAP kinase activation in cells exposed to a 60 Hz electromagnetic field. J Cell Biochem. 2003;90(6):1197–206. 10.1002/jcb.10704 14635193

[39] Patruno A, Pesce M, Grilli A, Speranza L, Franceschelli S, De Lutiis MA, et al mTOR Activation by PI3K/Akt and ERK Signaling in Short ELF-EMF Exposed Human Keratinocytes. Plos One. 2015;10(10). 10.1371/journal.pone.0139644 PMC4592237 26431550

[40] Urnukhsaikhan E, Cho H, Mishig-Ochir T, Seo YK, Park JK. Pulsed electromagnetic fields promote survival and neuronal differentiation of human BM-MSCs. Life Sci. 2016;151:130–8. 10.1016/j.lfs.2016.02.066 26898125

[41] Maredziak M, Tomaszewski K, Polinceusz P, Lewandowski D, Marycz K. Static magnetic field enhances the viability and proliferation rate of adipose tissue-derived mesenchymal stem cells potentially through activation of the phosphoinositide 3-kinase/Akt (PI3K/Akt) pathway. Electromagn Biol Med. 2017;36(1):45–54. 10.3109/15368378.2016.1149860 27367918

[42] Baek S, Quan X, Kim S, Lengner C, Park JK, Kim J. Electromagnetic Fields Mediate Efficient Cell Reprogramming into a Pluripotent State. Acs Nano. 2014;8(10):10125–38. 10.1021/nn502923s 25248035

[43] Costin GE, Birlea SA, Norris DA. Trends in Wound Repair: Cellular and Molecular Basis of Regenerative Therapy Using Electromagnetic Fields. Curr Mol Med. 2012;12(1):14–26. 22082478 10.2174/156652412798376143

[44] Cane V, Botti P, Soana S. Pulsed magnetic fields improve osteoblast activity during the repair of an experimental osseous defect. J Orthop Res. 1993;11(5):664–70. 10.1002/jor.1100110508 8410466

[45] Ahmadian S, Zarchi SR, Bolouri B. Effects of extremely-low-frequency pulsed electromagnetic fields on collagen synthesis in rat skin. Biotechnol Appl Biochem. 2006;43(Pt 2):71–5. 10.1042/BA20050086 16162095

[46] Feychting M, Ahlbom A. Magnetic-Fields and Cancer in Children Residing near Swedish High-Voltage Power-Lines. American Journal of Epidemiology. 1993;138(7):467–81. 8213751 10.1093/oxfordjournals.aje.a116881

[47] Savitz DA, Wachtel H, Barnes FA, John EM, Tvrdik JG. Case-Control Study of Childhood-Cancer and Exposure to 60-Hz Magnetic-Fields. American Journal of Epidemiology. 1988;128(1):21–38. 3164167 10.1093/oxfordjournals.aje.a114943

