# Supplementary figures and images for: A 60 Hz uniform electromagnetic field promotes human cell proliferation by decreasing intracellular reactive oxygen species levels
Source: PLoS One. 2018 Jul 16;13(7):e0199753. doi: 10.1371/journal.pone.0199753 (PMC6047776; doi:10.1371/journal.pone.0199753)

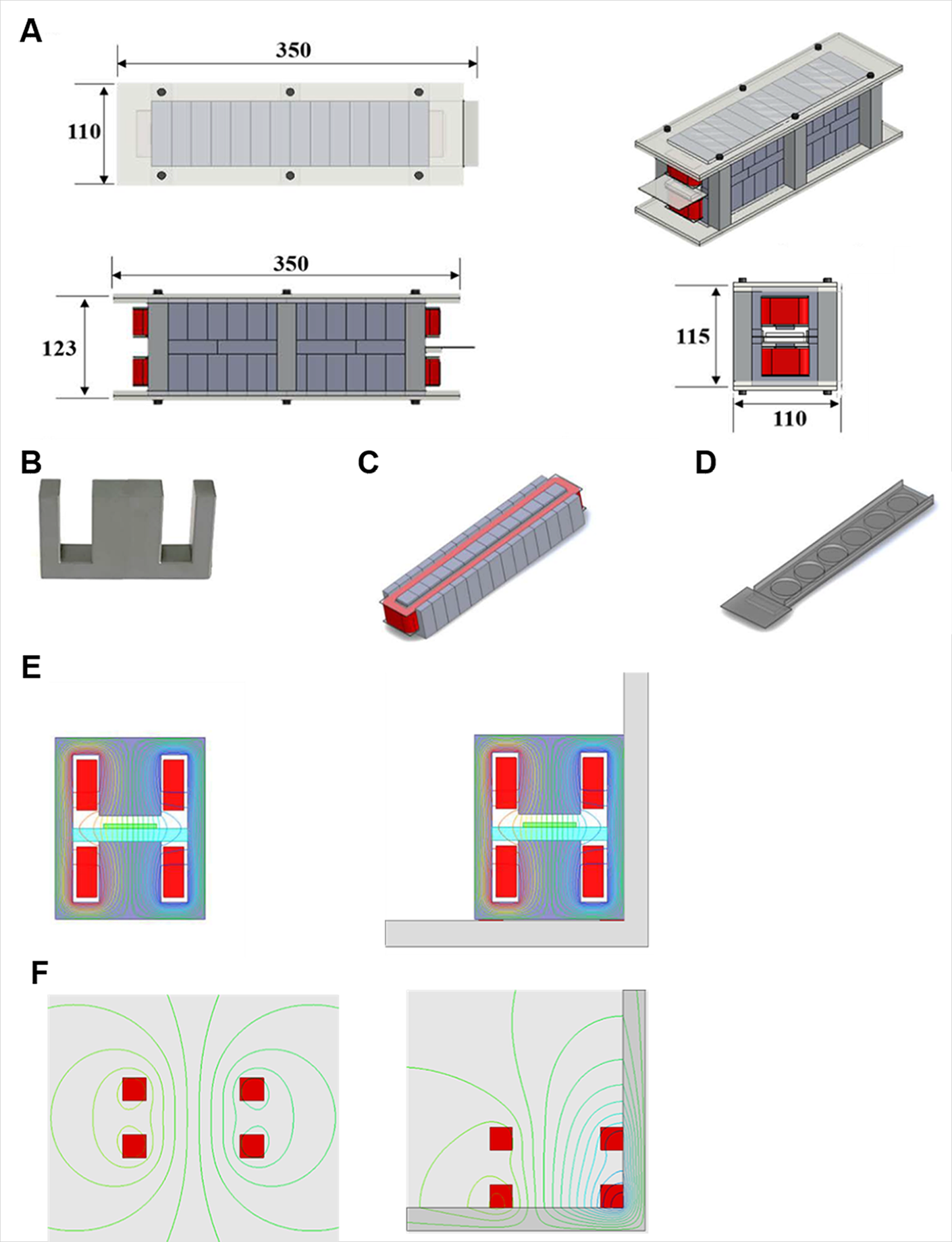

Supplement: S1 Fig — (A) Dimensions (mm) of the core device. (B) E-shaped ferrite core. (C) Bottom parts of the ELF-EMF device. (D) Plate for multiple dishes. (E) Variations of the flux line generated by the closed-type device in an incubator. (F) Variations of the flux line generated by the open-type device in an incubator. (TIF) [file pone.0199753.s001.tif]

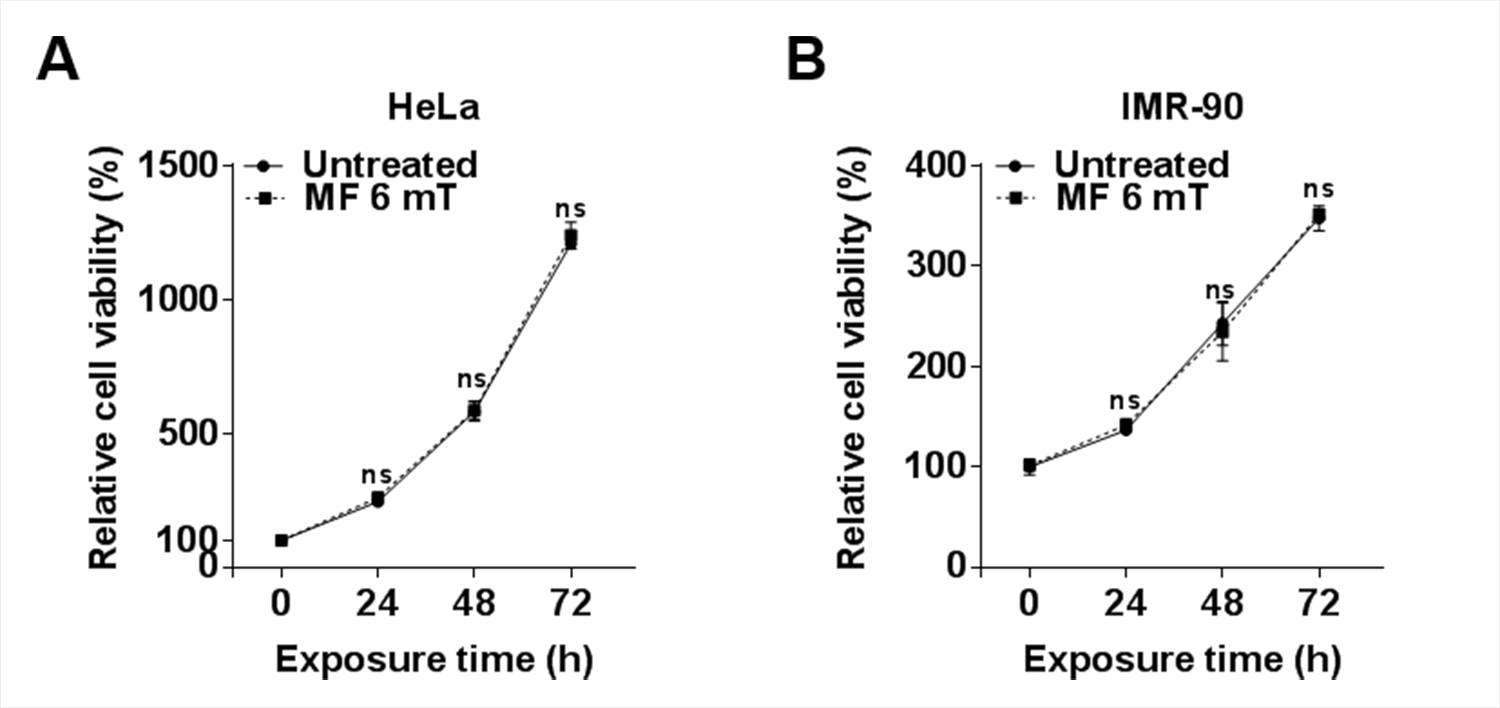

Supplement: S2 Fig — (A, B) HeLa and IMR-90 cells were exposed to an ELF-EMF of 6 mT for 30 min with 30 min intervals 8 times per day for 3 days and then cell viability was assessed by MTT assays every 24 h for 3 days. Cell viability was evaluated as a percentage relative to the viability of unexposed cells (0 h). Values are presented as the mean ± SD (n = 3) and P-values were determined by two-way ANOVA with the Bonferroni correction. P > 0.05 was considered statistically not significant (ns). (TIF) [file pone.0199753.s002.tif]

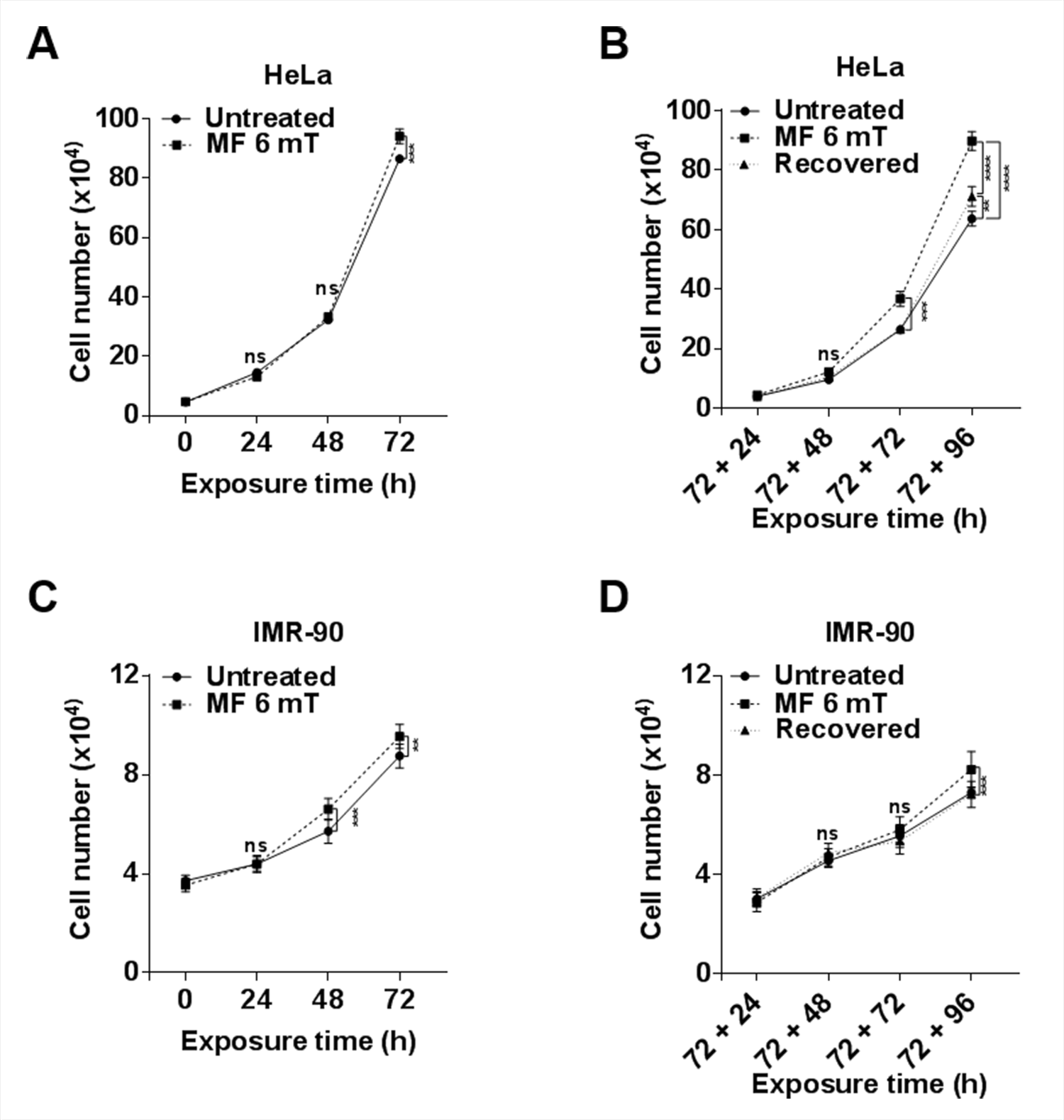

Supplement: S3 Fig — (A, C) HeLa and IMR-90 cells were continuously exposed to an EMF of 6 mT for up to 72 h. Cell number was counted every 24 h with a hemocytometer. (B, D) After 72 h of exposure to the EMF, HeLa and IMR-90 cells were detached and further subcultured in a uniform ELF-EMF of the same strength for up to 96 h. For the recovered group, cells exposed for 72 h were detached and subcultured without any further EMF exposure. In each group, cell number was counted every 24 h with a hemocytometer. Data were plotted as the mean ± SEM (n = 7). P-values were determined by two-way ANOVA with the Bonferroni correction. Values of *P < 0.05, **P < 0.01, ***P < 0.001, and ****P < 0.0001 were considered statistically significant, and P > 0.05 was considered statistically not significant (ns). (TIF) [file pone.0199753.s003.tif]

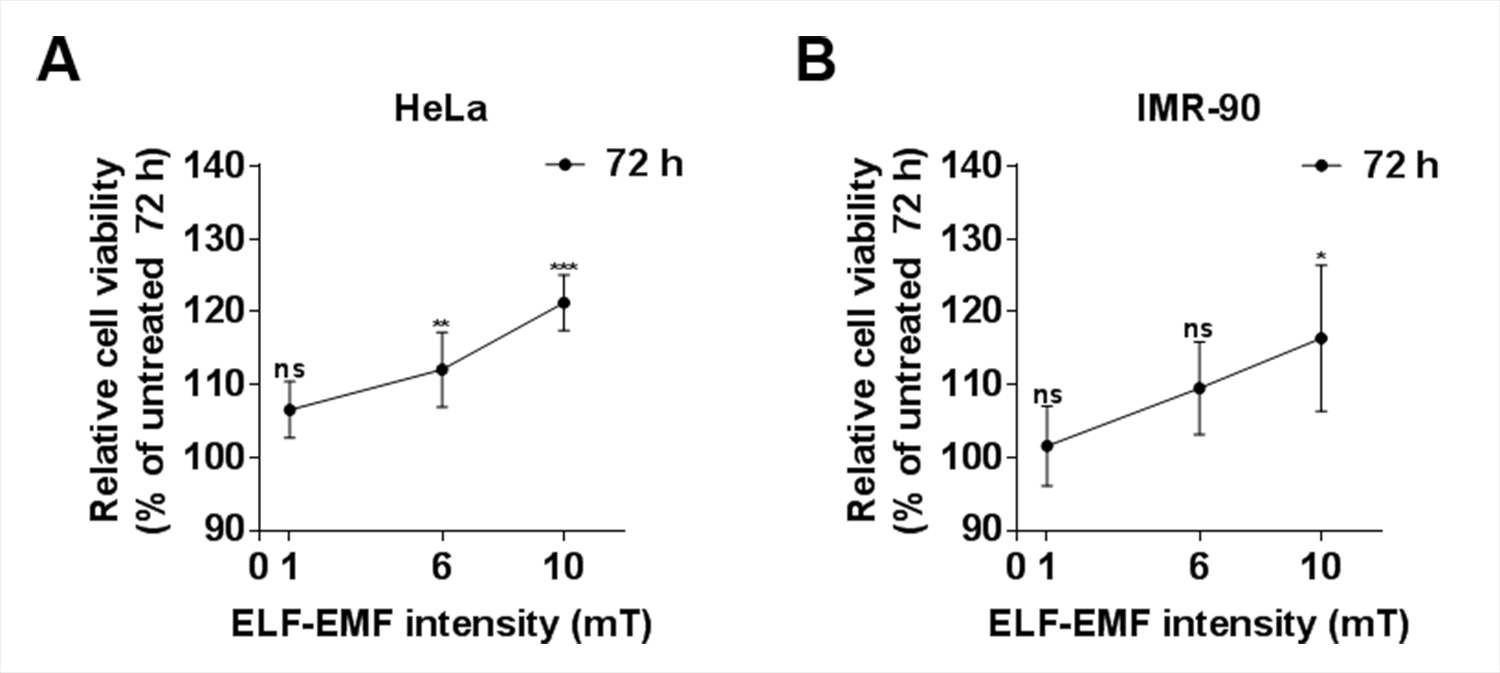

Supplement: S4 Fig — (A) HeLa and (B) IMR-90 cells were exposed to an EMF at 1, 6, and 10 mT for 72 h. Cell viability was assessed by MTT assays after a 72 h exposure. Relative cell viability (the viability of exposed cells relative to unexposed cells) of an EMF at 1, 6, and 10 mT was plotted as the mean ± SD (n = 3) and P-values were determined by two-way ANOVA with the Bonferroni correction. Values of *P < 0.05, **P < 0.01, ***P < 0.001, and ****P < 0.0001 were considered statistically significant, and P > 0.05 was considered statistically not significant (ns). (TIF) [file pone.0199753.s004.tif]
